# Private retail drug shops: what they are, how they operate, and implications for health care delivery in rural Uganda

**DOI:** 10.1186/s12913-018-3343-z

**Published:** 2018-07-09

**Authors:** Chrispus Mayora, Freddy Eric Kitutu, Ngianga-Bakwin Kandala, Elizabeth Ekirapa-Kiracho, Stefan Swartling Peterson, Henry Wamani

**Affiliations:** 1 School of Public Health, University of Witwatersrand, 27 St. Andrews Road, Parktown, Johannesburg, 2193 South Africa; 2 Department of Pharmacy, Makerere University College of Health Sciences, P.O. Box 7062, Kampala, Uganda; 3 Department of Women’s and Children’s Health, International Maternal and Child Health, Uppsala University, SE-751 85 Uppsala, Sweden; 4 Department of Mathematics, Physics and Electrical Engineering, Faculty of Engineering and Environment, Northumbria University, Newcastle upon Tyne, NE1 8ST UK; 5 Department of Health Policy Planning and Management, Makerere University School of Public Health, PO Box 7072, Kampala, Uganda; 6 Karolinska Institutet, Department of Public Health Sciences, Health System and Policy Research Group, SE-171 77 Stockholm, Sweden; 7 Department of Community Health and Behavioral Sciences, Makerere University School of Public Health, PO Box 7072, Kampala, Uganda

**Keywords:** Private sector, Retail market, Drug shops, Under-five children, Health care

## Abstract

**Background:**

Retail drug shops play a significant role in managing pediatric fevers in rural areas in Uganda. Targeted interventions to improve drug seller practices require understanding of the retail drug shop market and motivations that influence practices. This study aimed at describing the operational environment in relation to the Uganda National Drug Authority guidelines for setup of drug shops; characteristics, and dispensing practices of private retail drug shops in managing febrile conditions among under-five children in rural western Uganda.

**Methods:**

Cross sectional survey of 74 registered drug shops, observation checklist, and 428 exit interviews using a semi-structured questionnaire with care-seekers of children under five years of age, who sought care at drug shops during the survey period. The survey was conducted in Mbarara and Bushenyi districts, South Western Uganda, in May 2013.

**Results:**

Up to 90 and 79% of surveyed drug shops in Mbarara and Bushenyi, largely operate in premises that meet National Drug Authority requirements for operational suitability and ensuring medicines safety and quality. Drug shop attendants had some health or medical related training with 60% in Mbarara and 59% in Bushenyi being nurses or midwives. The rest were clinical officers, pharmacists. The most commonly stocked medicines at drug shops were Paracetamol, Quinine, Cough syrup, ORS/Zinc, Amoxicillin syrup, Septrin® syrup, Artemisinin-based combination therapies, and multivitamins, among others. Decisions on what medicines to stock were influenced by among others: recommended medicines from Ministry of Health, consumer demand, most profitable medicines, and seasonal disease patterns. Dispensing decisions were influenced by: prescriptions presented by client, patients’ finances, and patient preferences, among others. Most drug shops surveyed had clinical guidelines, iCCM guidelines, malaria and diarrhea treatment algorithms and charts as recommended by the Ministry of Health. Some drug shops offered additional services such as immunization and sold non-medical goods, as a mechanism for diversification.

**Conclusion:**

Most drug shops premises adhered to the recommended guidelines. Market factors, including client demand and preferences, pricing and profitability, and seasonality largely influenced dispensing and stocking practices. Improving retail drug shop practices and quality of services, requires designing and implementing both supply-side and demand side strategies.

**Electronic supplementary material:**

The online version of this article (10.1186/s12913-018-3343-z) contains supplementary material, which is available to authorized users.

## Background

Private retail drug shops play a significant role in health service delivery in low and middle income countries. Drug shops are small ‘walk-in’ health care shops that sell over-the-counter drugs. Sometimes drug shops sell other products other than medicines. A number of studies have indicated that in many low and middle income countries, retail drug shops are critical in providing health care services, and more so for common childhood illnesses [1]. In Togo for example, only less than 20% of under-five sick children visit the health facility, and up to 83% are managed at home using medicines procured from the retail market [2]. In Nigeria, more than 60% of malaria patients obtained their medication through patent medicine vendors (PMVs) – an equivalent of drug sellers [3, 4]. In Uganda and Tanzania, a sizeable amount of antimalarials are accessed through the retail sector market [5, 6]. Between 40 and 70% of pediatric fevers in Uganda are managed at retail drug shops, especially in rural areas. Retail drug shops are utilized as first points of call for health care for sick children especially in rural and geographically constrained communities [7–9]. The evidence on the extent of utilization of private retail sector by different socioeconomic groups is mixed, and largely context driven. There are reported variations in utilization of retail drug shops across and within countries. However, in urban areas, private retail sector services were more likely to be utilized by higher socioeconomic groups, compared to rural locations, where the private retail sector is commonly used by the lower socioeconomic groups [10].

The prominence of retail drug shops owes itself to existing constraints in access to public health services, including: long distances, household poverty, and limited or even lack of well-functioning public health facilities in some geographical areas – characterized by inadequate health workers, drug stock outs, and perceived poor quality services [6, 9, 11–16]. Retail drug shops are perceived to be accessible, convenient, flexible in terms of operating hours, offer credit facilities, trusted by communities, and are perceived to provide services at lower costs [15, 17–19]. However, there are also concerns about the quality of services retail drug shops provide. For example, there are reports that most drug shop attendants are unqualified and lack adequate knowledge and capacity to appropriately identify illnesses and offer appropriate treatment for illnesses, stocking and dispensing substandard, counterfeit, or even expired drugs, among others [1, 9, 20].

Notwithstanding the reported challenges, retail drug shops are a potential way to increase coverage of health services and achieve universal health coverage (UHC). Retail providers could relieve pressure off a health care system that is struggling to deliver comprehensive health services amidst resource challenges. The Global Fund’s Affordable Medicines Facility Malaria (AMFm) conducted pilot program in eight countries, including Uganda, to test the feasibility of subsidizing medicines through the private sector on availability, affordability and access to medicines. Results from this AMFm pilot, indicated that, indeed, subsidizing medicines provided through the private sector can improve availability, affordability, and access, to medicines particularly for childhood conditions [21].

In Uganda, drug shops are recognized within the health care system as part of the private for profit (PFP) sector. Retail drug shops are registered, licensed and regulated by the national drug authority (NDA). Drug shops are classified as ‘C’ (permitted to sell a restricted list of medicines, including some antimicrobial formulations). Although drug shops are expected to be registered and licensed before they start operations [22], this sometimes is not the case. In addition, while the NDA Act stipulates the type of cadre supposed to run the drug shops, often times, there is lack of consistent enforcement and compliance. Sometimes drug shops to present qualified personnel when seeking for accreditation and thereafter recruit unqualified ones once licensure processes are complete. It is therefore not uncommon to find untrained staff working in drug shops in addition to stocking medicines that are not recommended for those classes or drug shops.

While retail drug shops have proliferated in the recent past especially in low and middle income countries, not much attention has been paid to them [23]. In Uganda, studies have focused more on understanding the role of retail drug shops, quality of services provided, feasibility of using rapid diagnostics at drug shops, delivering family planning products through drug shops, among others [9, 24–28]. However, in Uganda, there is limited information on the structure and operational characteristics of the retail drug shop market. Yet, this would provide an important starting point for designing feasible interventions aimed at improving the potential of retail drug shops to provide appropriate and quality services. The aim of this paper was to characterize private retail drug shops, their operational environment, as well as elicit factors that influence their day-to-day decisions in the management of febrile conditions among children less than five years old in rural South Western Uganda.

## Methods

This study used data collected as part of the baseline survey conducted as part of a research project implemented by Makerere University School of Public Health (MakSPH), entitled – “ACCESS and EXCESS, EQUITY and INFORMATION: Point of Care Diagnostics and Pre-packaged Subsidized Drugs for Integrated Fever Management for Malaria, Pneumonia and Diarrhea in Children at PRIVATE SECTOR Drug Shops in Uganda” In brief, the research project aimed to test the feasibility and effect of implementing integrated community case management (iCCM) for childhood illnesses, on pediatric care, at licensed retail drug shops in a low malaria transmission setting in Uganda. The baseline survey was conducted in May 2013, among licensed and operational retail drug shops in two districts of Mbarara and Bushenyi, in South Western Uganda. More detailed information about the research project has been published elsewhere [19, 28]. In the study area, health services are provided through public health facilities, private-not-for profit facilities, private hospitals and clinics, pharmacies, retail drug shops, and sometimes the traditional medicine providers. The districts health system is superintended by the district health teams (DHT) with the district health officer (DHO) as the overall technical head. The registration, licensure, accreditation, and regulation of retail drug shops is the mandate of the National Drugs Authority (NDA) which delegates authority to the District Drugs Inspector of each district (DDI). Among others, applicants intending to open up drug shops are required to present certificates of qualification for intending in-charges, letter of commitment from the in-charge, suitability of the premises, among others [22]. In terms of epidemiology, the prevalence of malaria among children (0–59 months) in this region (South Western Uganda) is reported to be 5.7% (by Rapid Diagnostic Tests (RDT)) and 4.1% (by Blood Smear Microscopy (BSM)) which is significantly lower than the national averages of 30% (by RDT) and 19% (BSM), among the same group. The same national malaria survey indicates that malaria prevalence was high in rural areas (10.1% for Urban versus 32.4% for rural, by RDT, compared with 6.3% for urban versus 22.3% for rural, by BSM) compared to the urban counterparts [29]. The study area is a rural setting with socioeconomic and infrastructural challenges of access to formal health care services. Therefore, retail drug shops have emerged to fill the gap in availability health care facilities within reach.

### Sampling and sample size

A total of 217 drug shops in the study area were assessed for eligibility. The list was obtained from the National Drug Authority (NDA) register available at the District Drug Inspector (DDI). More detail on assessing the drug shops for inclusion in research project has been described elsewhere by kitutu et al. [28]. The eligibility for participation required that drug shops had to be in the rural area, functional, dealing with human drugs, and acceptance to participate in the survey. In Mbarara, out of 152 drug shops, only 61 drug shops met the inclusion criteria, but 40 accepted to participate in the survey. In Bushenyi, 65 drug shops, were eligible, and data was collected from 34 drug shops. Therefore, a total of 74 registered, licensed, and functional retail drug shops were included in this study. During the survey, drug shop attendants were interviewed. In addition to the drug shop attendants’ interviews, exit interviews were conducted with caretakers of children under five years of age who had come to purchase medicines from the drug shop during the study period. Taking drug shops as clusters, and using the Bennet cluster sampling method [30], 428 exit interviews conducted at 24 clusters were estimated. However, only 12 and 10 clusters in Mbarara and Bushenyi Districts, respectively, were covered during the exit interviews. At each selected drug shop, an average of 20 exit interviews were conducted.

### Data collection

Data for this study was collected at baseline (May, 2013) through a drug shop survey where drug shop attendants were interviewed using a semi-structured questionnaire that collected information on drug shop characteristics and practices: ownership, training of attendants, commonly stocked medicines, stocking, prescription and dispensing decisions, among others (see Additional file 1). Respondents in the exit interviews were caretakers of children under-five years of age that came to seek care or to purchase medicines for febrile conditions. These were contacted immediately as they left the drug shop and interviewed at a convenient place within the vicinity of the drug shop, so as not to allow drug seller’s interference. The structured exit interview questionnaire specifically elicited additional information on client characteristics, drug shop diagnostic, prescription, and treatment practices, as well as client decisions around the drug shop use, nature of illness, onset of illness and time taken to make a decision to seek care, among others (see Additional file 2). All data collection tools were translated into, and interviews conducted in Runyankore, the local language used in the study area. Research assistants were selected from the study districts (local area), because it was necessary to have individuals who spoke and understood both local language and English. For exit interviews, interviewers were stationed at locations near drug shops all through the exit interview period. Exit interview respondents were recruited consecutively after they exited the drug shops, and interviewed if they accepted to participate. Interviewers also observed or checked the medicines provided to clients to verify the responses to posed questions.

### Analysis and quality control

Research assistants were trained for two days and a pilot conducted before embarking on full data collection. All data were checked for completeness and accuracy by the group supervisor, at the end of every data collection day. Data was double-entered in epi data (http://www.epidata.dk) and was analyzed using STATA version 12 (http://www.stata.com). All data entry, cleaning and transfer processes were supervised by the lead author for purposes of quality control. This study adopted first level descriptive analysis, to generate and report results in terms of means and proportions of variables. Appropriateness of treatment was assessed through exit interviews based on reported symptoms and medicines provided. It is common practice for care-seekers not to come with sick children to the drug shop, and instead drug sellers rely on description of symptoms by the caregiver. Clients were asked at exit, what diagnostic processes had been conducted, and in some cases what symptoms had been reported. The interviewers then checked medicines they had been provided including their dosages, frequency of use, and other indications. The reported symptoms and medicines dispensed were then compared with national and WHO guidelines [31, 32]. Particular variables or characteristics under study were analyzed and compared across the two study groups (districts) using a chi-square test, to understand if the study characteristics differed significantly across the two study groups. We presented *p*-values from the corresponding chi-square tests, with a p-value of < 0.05 being considered statistically significant.

### Ethical considerations

This study used data collected through a baseline survey conducted as part of a research project implemented by MakSPH whose ethical approval was obtained from the Uganda National Council for Science and Technology (UNCST) (#HS1385), and the MakSPH Higher Degrees Research Ethics Committee (#IRB00011353). In addition, this particular study was approved by the University of Witwatersrand Human Research Ethics Committee (HREC) (#M151016). Further permission was obtained from the National Drugs Authority (NDA) and the Local Government authorities for the two districts. Written consent was obtained from drug sellers and exit clients who participated in this study.

## Results

### Characterizing drug sellers and care-seekers of children under five years of age who use retail drug shops

We profiled the drug shop attendants and the care-seekers of under-five children who came to seek care and purchase medicines at the retail drug shop during the survey period, based on selected socioeconomic indicators, such as training, education status of clients, and incomes. Results showed that the real owners of drug shops attended to 26 and 65% of the drug shops surveyed in Mbarara and Bushenyi respectively (Table 1). Most drug shop surveyed, were attended to by nurses or midwives, and the rest were nursing assistants, laboratory technicians, pharmacists, and clinical officers.

**Table 1:** Characterizing drug shop attendants and care-seekers of children under five years seeking care from retail drug shops

Drug seller Characteristics	Mbarara (*N* = 40)	Bushenyi (*N* = 34)			
	n (%)	n (%)	*P*-value*		
Who attends to the drug shop					
Owner	10 (25.6)	22 (64.7)	< 0.00		
Highest level of training for drug shop attendant interviewed					
Clinical Officer	8 (20.0)	5 (14.7)			
Nurse/Midwife	24 (60.0)	20 (58.8)	0.73		
Others*	8 (20.0)	9 (26.4)			
Monthly salary earnings for drug shop attendants interviewed					
USD 9 – USD 44	24 (80.0)	34 (100.0)			
USD 45 – USD 88	16 (20.0)	0 (0.00	< 0.00		
Care-seekers’ characteristics (Exit interviews)	Mbarara (*N* = 212)		Bushenyi (*N* = 216)		
	n (%)	95% CI (in %)	n (%)	95% CI (in %)	*P*-value*
Highest level of education of care-seeker					
University	2 (0.95	[0.2, 3.7]	4 (1.8)	[0.69, 4.8]	
Tertiary	2 (0.9)	[0.2, 3.7]	13 (6.0)	[3.51, 10.1]	
High School	7 (3.3)	[1.5, 6.8]	7 (3.2)	[1.54, 6.6]	
O-Level	59 (28.1)	[22.4, 34.5	76 (35.1)	[29.07, 41.8]	< 0.00
Primary	123 (58.5)	[51.7, 65.0]	109 (50.4)	[43.79, 57.1]	
None	17 (8.1)	[5.0, 12.6]	7 (3.2)	[1.54, 6.6]	
Employment status of care-seeker	(*n* = 209)		(*n* = 214)		
Unemployed	8 (3.8)	[1.9, 7.4]	40 (18.6)	[13.92, 24.4]	
Housewife	25 (11.9)	[8.1, 17.0]	53 (24.6)	[19.32, 30.8]	< 0.00
Self-employed	92 (43.8)	[37.2, 50.6]	87 (40.4)	[34.07, 47.1]	
Civil Servant	5 (2.3)	[0.9, 5.6]	5 (2.3)	[0.96, 5.4]	
Others	80 (38.1)	[31.7, 44.8]	29 (13.4)	[9.51, 18.7]	

*P values presented are derived from chi-square, and it is comparing indicators across the two study groups (districts)

### Retail drug shop operational environment and other services provided

The quality of medicines and other services provided by retail drug shops is partly related to the physical environment of the drug shops. The National Drug Authority (NDA) Act recommends that medicine stores should be free from moisture, direct sunlight, and other exposures. Medicine storage and exposure to moisture influences the quality and efficacy of medicines. This study investigated – through interviews and using an observational checklist – the physical conditions under which retail drug shops operated. In Table 2, most drug shops adhered to the guidelines specified in the NDA licensing guidelines. Most surveyed drug shops had storage area for medicines secured against thefts, direct sunlight, and moisture, had ceilings and controlled room temperatures, among others, although additional improvements could be made. Beyond, the sale and dispensing of medicines, some (12%) retail drug shops in Bushenyi offered immunization services, over two-third of drug shops in both districts offered Vitamin A supplementation, while others sold deworming, soft drinks, toiletries, cosmetics products, mobile telephone credit, among others.

**Table 2:** Operational environment and other services provided by retail drug shops

	Mbarara District (*n* = 40)	Bushenyi District (*n* = 34)	
Variables at baseline (*n* = 74)	Number of drug shops (%)	Number of drug shops (%)	*P*-value
Drug shop environment/ conditions			
There exists a ceiling to control room temperature	40 (100.0)	28 (82.3)	< 0.00*
There are windows that can control aeration	29 (72.5)	15 (44.1)	< 0.01*
No direct sunlight to area for keeping medicines	40 (100.0)	33 (97.0)	0.27
Medicine store free from moisture from leaks	37 (92.5)	27 (79.4)	0.10
Premises can be secured to prevent theft	38 (95.0)	32 (94.1)	0.86
Other services provided by drug shops			
Immunization Services	0 (0.0)	4 (11.7)	0.02*
Vitamin A supplementation	31 (77.5)	23 (67.6)	0.34
Deworming	4 (10.0)	5 (14.7)	0.53
Reporting adverse drug reactions	18 (45.0)	12 (35.2)	0.39
Presence of treatment guidelines			
Uganda Clinical Guidelines	16 (40.0)	22 (64.7)	0.03*
iCCM Guidelines	6 (15.00	9 (26.4)	0.22
Malaria treatment algorithms/charts	17 (42.5)	15 (44.1)	0.88
Pneumonia treatment algorithms /charts	2 (5.0)	5 (14.4)	0.15
Diarrhea treatment algorithms/charts	12 (30.0)	15 (44.12	0.20
Other products traded at drug shops			
Soft drinks	4 (10.0)	1 (2.9)	0.22
Toiletries	4 (10.0)	2 (5.8)	0.51
Household goods	1 (2.5)	0 (0.0)	0.35
Mobile airtime	3 (7.5)	1 (2.94	0.38
Cosmetics	1 (2.5)	0 (0.0)	0.35
Food items	0 (0.0)	2 (5.8)	0.12

### Common signs and symptoms reported at drug shops

Based on results from the exit interviews, the common signs and symptoms that were reported at drug shops by care-seekers of children under five years of age, for which they came to seek medication were: fever (239/428 or 56%), cough (281/428 or 66%), rapid or difficult breathing (86/428 or 20%), and diarrhea (114/428 or 27%). Other common childhood illness signs and symptoms reported at retail drug shops accounted for 101/428 (24%).

### Common medicine stocks and stocking decisions

**Fig. 1:**
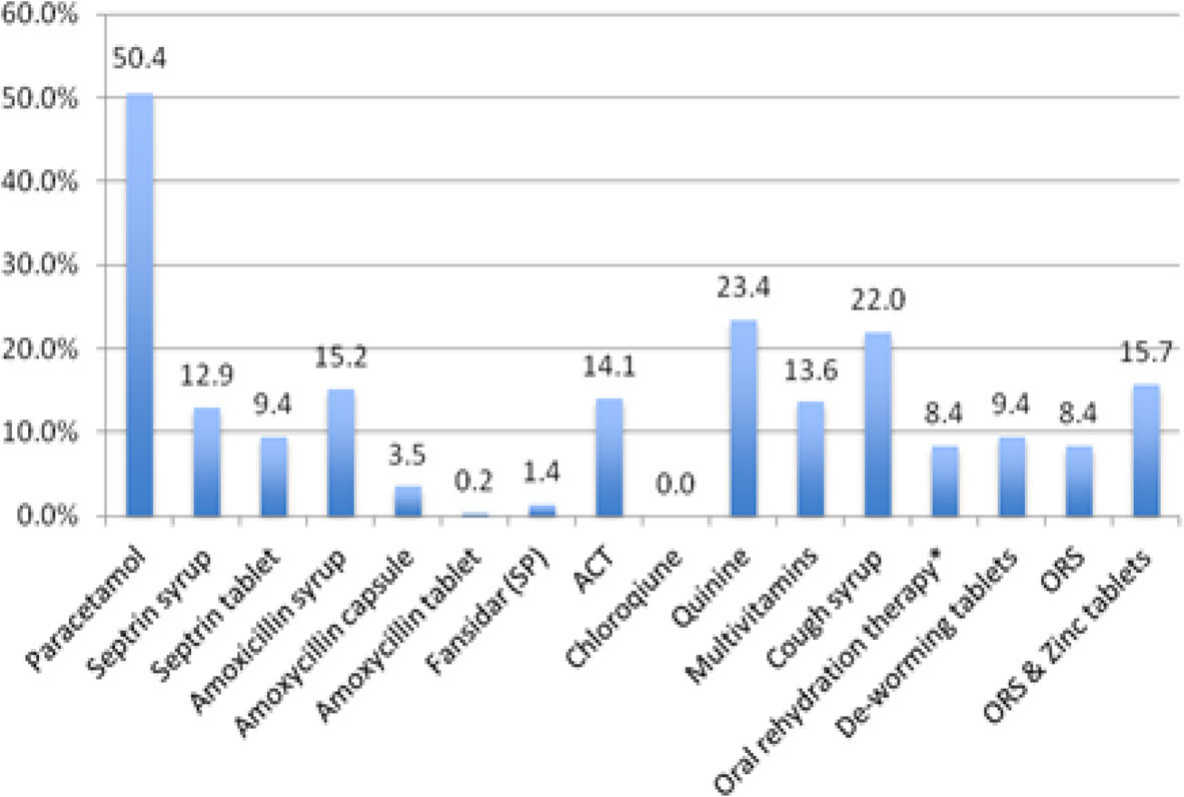
Commonly stocked medicines by retail drug shops at time of survey. *ACT = Artemisinin-based Combination Therapies, SP=Fansidar, ORS=Oral Rehydration Solution.

From drug shop attendants’ interviews and observations, this study found that the common medicines stocked by retail drug shops included Paracetamol, cough syrup, Amoxicillin dry syrup, Quinine, ORS/Zinc, and Artemisinin-based Combination Therapies (ACTs), among others **(**Fig. 1). Drugs shop attendants were asked to elicit the factors they put under consideration when deciding what medicines to stock at a particular point in time. The main factors were reported to be, consideration of Ministry of health recommended drugs (54%), medicines commonly demanded by clients (70%), most profitable medicines (26%), and seasonal disease patterns (30%) and the influence of sales agents from pharmaceutical companies (19%) who sometimes move around communities and small local towns selling medicines and other pharmaceutical products.

### Drug shop attendants’ dispensing decisions

**Fig. 2:**
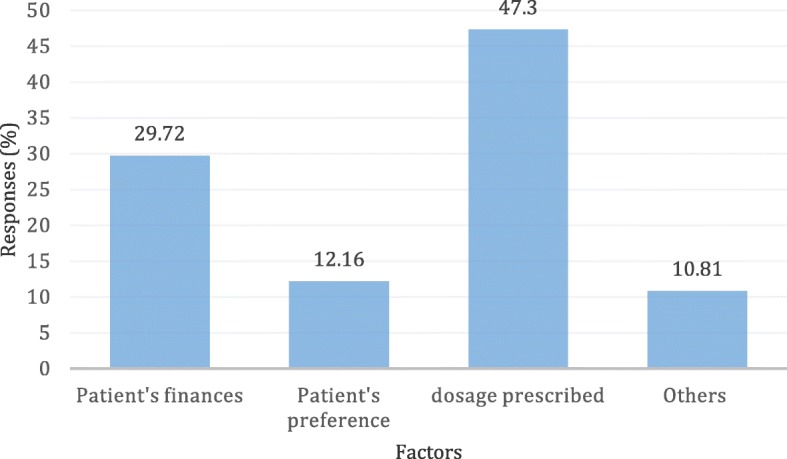
Factors influencing drug sellers’ dispensing practices

Drug sellers’ decisions about what medicines to dispense, were mainly influenced by among others; prescriptions that some clients come with (47%) (Prescriptions are sometimes provided at public health facilities), amount of funds that a client comes with at the drug shop (30%) and client’s patient preferences for particular medicines (12%), among others **(**Fig. 2**)**. However, drugs shop attendants were asked what decisions they made in cases where clients did not have enough finances to procure the recommended medicines and dosages, drug sellers reportedly offered additional medicines on credit (69%), offered cheap alternatives (18%), or referred the client elsewhere (27%) (Results not shown).

### Appropriateness of treatment practices by retail drug shops

We used exit interviews to understand treatment practices of drug sellers, particularly the use of appropriate diagnostics and prescription of medicines. We found limited use of diagnostics – malaria rapid diagnostic tests (RDTs), respiratory count timers, and thermometers. Only about 21% care-seekers who brought sick children reported having been diagnosed with RDTs, 21% with thermometer, and no child had their breathing rate counted using a respiratory timer. Relating the symptoms reported by care-seekers and medicines given at client exit, based on the Uganda iCCM Guidelines [32], and World Health Organization Guidelines on rationale medicines use [31], the study found only 4% had received correct antibiotics, 39% had received antimalarials (ACTs), and 57% had received correct diarrhea treatment. A number of retail drug shops surveyed however, had clinical guidelines, iCCM guidelines, malaria and diarrhea treatment algorithms and charts.

## Discussion

In this study, we sought to characterize the private retail drug shops, explore their operational environment, as well as elicit factors that influence their day-to-day decisions in the management of febrile conditions among children less than five years old in rural South Western Uganda. Although the study focused on one segment of the retail health market – the registered retail drug shops – the results presented have indeed revealed interesting features of the retail drug shops, which are significant for policy and programming. The main findings indicate that most surveyed retail drug shop premises met the NDA requirements of setting up premises that deal in medicines. These guidelines were established to assure medicines safety and quality. Drug sellers had health-related qualification with majority being nurses or midwifes. The most commonly reported and managed childhood illness signs and symptoms at drug shops were fever, cough, rapid or difficult breathing, and diarrhea. It was also found that retail drug shops commonly stocked Paracetamol, Quinine, Cough syrup, ORS/Zinc, Amoxicillin dry syrup, Septrin® syrup, Artemisinin-based Combination Therapies, and multivitamins. Decisions on what medicines to stock were influenced by among others, Ministry of Health recommended medicines, medicines demand, most profitable medicines, and seasonal disease patterns. Relatedly, dispensing decisions were influenced by among others: prescriptions presented by the client, patients’ finances, and patient preferences**.** In response to clients with insufficient resources, drug sellers either offered credit depending on the relationship and trust cultivated overtime, or offered cheaper product alternatives.

Our finding that most of drug sellers (attendants) had health related qualifications – with the common qualification being nurses or midwifes, and clinicians, could be related to the fact that only registered drug shops were enrolled for the survey, owing to the legal and policy challenges that would arise by engaging un-registered and hence unauthorized entities. It is not uncommon during registration and licensure to ensure drug sellers with appropriate qualification are responsible, otherwise a license may never be granted. It is relatively complex to enforce such requirements for unregistered drug shops that are essentially operating illegally. It is important however to note that while the new NDA Professional Licensing guidelines 2018 [33] allow nurses, midwives, and Pharmacy Technicians or dispensers to attend to human drug shops, within the context of Uganda’s medical training system, nurses and midwives had no special training that would aid them to prescribe medicines. For long, it had been Pharmacy Technicians who were allowed these roles, but with the current health workforce challenges that characterize Uganda, manifesting in chronic shortages for human resources for health, this may not be possible. The training and skills of drug sellers or attendants has a significant bearing on the quality of services offered at retail drug shops. Mbonye and colleagues, found professional qualification of a provider to be highly associated prescriptive practice, with lower cadre staff (nursing assistants and enrolled nurses) overprescribed antibiotic [34]. There are other studies that have reported gaps on training and skills of drug sellers [35, 36] However, with minimal complementary training and capacity building support for example through continuous training, monitoring and onsite support supervision, drug sellers could improve their treatment and dispensing practices and hence improve quality of care provided [9, 37, 38].

Our study found that most drug shop premises met the NDA requirements allowed for premises that operate drug shops. We found that most surveyed drug shops had secure drug storage, medicines were protected against moisture and sunlight, and drug shops operated under controlled room temperature. Appropriate conditions for storage and dispensing of medicines are critical in maintaining their quality and safety during their shelf life. We found that some drug shops provided additional services and products beyond medicines, including soft drinks, mobile phone credit, deworming, and immunization services. For the few drug shops that provided immunization services, additional storage capacities for vaccines (e.g. refrigeration and cold chain systems) were not available yet extremely necessary. However, it is important to note that the current NDA guidelines and policy framework does not allow retail drug shops to provide such services. Business diversification, the drug sellers argued, was a mechanism for business sustainability and mitigating high operational costs. The more diversified the business, the less likely that a drug shop would rely on one business line as source of business survival. Product and service diversification increases retail sales volumes and significantly reduces the average costs of production, with possibilities of transferring these cost cuttings to clients in form of reduced prices [39]. Aspects of incentives for business start-ups and operational costs in retail health markets, was investigated separately by the author and colleagues, and findings shall be shared in separate publication. It is important, however, that these additional lines of business are related to the mainstream service or products being offered. Drug shop operators must also balance this diversification agenda against its feasibility, risks, and limitations.

The most common childhood illnesses reported at the drug shops were found to be fevers, coughs, difficulty in breathing, and diarrhea. The current burden of childhood illnesses in Uganda, shows that malaria, acute respiratory illness and diarrhea are major contributors to mortality and morbidity for children under five years of age [29], and this is true for Sub-Saharan Africa (SSA). To achieve greater reductions in the burden of under-five mortality and improve child survival in Uganda, retail drug shops must be brought into perspective. Retail drug shop practices need to be improved. In this study, we found limited use of diagnostics – malaria RDTs, respiratory count timers, and thermometers. As starting point, there is need to improve diagnostic capacities of retail drug shops as a critical ingredient for appropriate treatment and care, and well in line with the current WHO “test and treat” policy [26, 40, 41].

The potential for drug shops to use RDTs and reduce the unnecessary prescription and use of antimalarials has been demonstrated by a number of studies conducted in many settings, including in Uganda [26, 42] [27, 28, 43]. The WHO “*test before treat policy*” now recommends parasitological confirmation of malaria before antimalarials are provided [44]. Indeed, the WHO Framework on malaria elimination under the Global Technical Strategy for Malaria 2016–2030, provides for creating an enabling environment for ensuring universal access to malaria prevention, diagnosis, and treatment [45]. Inappropriate treatment resulting from the limited application of diagnostics has been a consistent finding across a number of studies [46–49]. Inappropriate prescription and unnecessary use of antibiotics has been established to be associated with antimicrobial resistance (AMR) [50], which is currently a global public health challenge that must be addressed. The current Malaria Treatment Policy and the Uganda Malaria Control strategic Plan now recommend the use of RDTs at community level and in the private sector [51], and additional guidelines for using RDTs in private outlets in Uganda have been developed [52] . The Global Fund for HIV/AIDS, Malaria, and Tuberculosis (GFMAT) through the private sector co-funding mechanism, has also been supporting the private sector in improving the standards of malaria diagnosis and treatment in Uganda. However, there are important challenges such as training and supervision, waste management, streamlining the referral system from private outlets, surveillance, etc. that need to be considered [53]. The policy reforms being undertaken require careful planning and consideration of training, regulatory, supervisory, and other necessary capacities, coupled with necessary incentives for private outlets to improve their practices [26].

Our study found that prescription or dispensing decisions were not only related to existing diagnostic capacities, but were also influenced by other factors, including: the clients’ finances, prescriptions that patients came with, and client’s preferences. Wafula and colleagues, in a systematic review of drug shops in Sub-Saharan Africa, reported that, among others, client demand strongly influenced dispensing practices. They noted that most drug shops simply sold medicines that clients requested for or preferred, without necessarily following policy recommendations. The systematic review also reported that, rural drug shops provided credit facilities to their clients, in cases where they had no resources to afford the full dosages of medicines [54]. Similarly, Goel and colleagues, in their study of the behavior of retail pharmacies in developing countries, also reported that client demand or expectations as well as local regulatory factors strongly influenced drug selection and dispensing behavior [55]. Evidence from both our study and the literature, indicate that dispensing behavior is related to both demand and supply side factors, and thus, improving dispensing practices requires strategies or interventions on both the demand side and supply side of the market. For example, training, monitoring, supervision, and subsidies (supply side) are critical supply-side strategies. They alone, may not be sufficient in influencing dispensing behavior, rather, additional demand-side initiatives are necessary. Programs such as public health education and the provision of adequate information through mass media campaigns, may address issues of adverse effects of incomplete dosages, and the dangers of self-medication and prescription. Other demand-side strategies may include social marketing and the provision of demand-side subsidies. Demand-side strategies are aimed at influencing consumer behavior, preferences, and choices, and this would ultimately influence dispensing practice [54]. The design of any package of interventions and market incentives, must however recognize the broader health system effects that may likely emerge from those interventions, beyond the market where they are implemented [19].

Relatedly, drug shop attendants indicated that their medicine stocking decisions were influenced by: medicines on demand, ministry of health recommended, most profitable medicines, and seasonal dynamics. These factors have also been identified in studies elsewhere [56–58]. According to the NDA Act, drug shops are not expected to store and dispense antibiotics, except in exceptional circumstances where a drug shop is allowed to sell antibiotics especially if the drug shop is the only sources of such life-saving medicines for particular communities [22]. Our study however, found that amoxicillin (syrup and capsules) was one of the commonly stocked and dispensed medicines at surveyed drug shops because of its high demand. In many cases, drug shops that stored antibiotics did not display them on the shelf for fear of reprisals from drug inspectors, but would provide the client the same on demand. In addition, drug shops were still found with stocks of medicines such as quinine that are no longer recommended as first-line treatment for malaria. Surprisingly, the study found that most drug shops had clinical guidelines and regulations available, yet it was clear that many drug sellers were not in many cases adhering to them. It was not apparently clear whether it was a deficiency in enforcement, inadequacy in training, or rather an issue of incentives for adherence. Reflecting on elicited factors that influence treatment practices, private retail drug shops were likely to promote the practice of self-medication, especially in circumstances where client preferences, and prescriptions that come with, were major considerations in dispensing decisions. Incentive mechanisms that enable drug shops to adhere to rules could be established, including strong monitoring and supervision and deregistration in cases of inappropriate practices [59, 60].

Finally, this study profiled the drug shop clients socioeconomically to get a basic understanding of the type of clients who procure medicines from the retail drug shops and the related equity issues. This study found that care-seekers of children under five years of age who sought care from retail drug shops during the survey period were mostly those who are unemployed, self-employed and less educated. In Uganda, it is not uncommon to find strong relationships between these strata and lower socioeconomic quintiles. It is important to note that the poor are also the most vulnerable to the burden of ill-health. Thus, interventions aimed at improving retail drug shop practices were likely to benefit the poor in society and ensure improved equity in access to health care. However to achieve higher equity, a mix of interventions, including consumer economic empowerment, and a stronger regulatory regime could cushion consumers against possible market distortions and exploitation in the form of high prices and poor quality of products and services. In addition, there is need to establish the right incentives that will influence provider and consumer behavior towards outcomes that are in public interest [19] [61, 62].

This study however, had some limitations that need to be underscored, including the fact that only registered retail drug shops were included in the study. Yet, there are many unregistered drug shops that partly influence what happens in that market, and so the results reported can only be relevant to the extent of registered drug shops. Unlicensed outlets were excluded from the survey because it would have been ethically unacceptable to promote “outlets” that have selected to defy an existing regulatory regime. Secondly, the determination of appropriate treatment was based on responses from care-seekers during exit interviews. We asked the client what signs and symptoms they had reported to the drug seller, observed the medicines that had been provided, and also compared with clinical guidelines and WHO guidelines on promoting rational use of medicines, to generate an “appropriate treatment” outcome. This may have inherent flaws especially the reliance on signs and symptoms reported without due diagnosis for conclusiveness. We however endeavored to validate the response with the drug seller on reported symptoms of immediate previous client, after the exit interview.

## Conclusion

Retail drug shops were an important source of care for childhood conditions especially in rural and geographically constrained settings. Most retail drug shops’ physical environment met the guidelines established by the National Drugs Authority (NDA). Most Drug shop attendants were nurses, midwifes, clinical officers and some pharmacists. A physical environment in line with the NDA guidelines, coupled with some health related training by drug sellers, offer a starting point to improve the quality of services retail drug shops provide. Drug sellers’ stocking and dispensing practices were largely influenced by market factors including client demand, client finances, prices and profitability, client preferences, and seasonal factors. To ensure effectiveness, the factors influencing drug shop practices and care must be taken into account for any intervention or strategy. Mechanisms that influence the market demand and supply are necessary to improve drug seller practices. Strategies such as regulation, monitoring and supervision, as well empowering communities with information, and provision of subsidies may be apparent. Additional support in terms of provision of subsidized medicines and diagnostic technology may go a long way in reducing prices of medicines and services provided by retail drug shops, for the ultimate benefit of the consumers. Beyond subsidization, however, retail drugs shops need to be, retail drug shops need to be supported through implementation of capacity building initiatives, such as periodic trainings of drug sellers to enhance dispensing capabilities.

## Additional files

Additional file 1: Drugshop Survey Tool This is the questionnaire that was administered to drug sellers to generate information on the drug shop market. (DOC 216 kb).

Additional file 2: Client Exit Interview Tool This is the questionnaire that was administered to drug shop clients at their exit from the drug shop when they came to purchase medicines or drugs for under-five children, during the study period. (DOC 520 kb).

## Abbreviations

ACTs: Artemisinin-based combination therapy
AMFm: Affordable Medicines Facility – Malaria
AMR: Antimicrobial Resistance
BSM: Blood smear microscopy
DDI: District Drug Inspector
DHO: District Health Officer
DHT: District Health Team;
GFMAT: Global Fund for HIV/AIDS, Malaria, and Tuberculosis
HREC: Human Research Ethics Committee
iCCM: Integrated Community case management
LICs: Low-income countries
MakSPH: Makerere University School of Public Health
mRDTs: Malaria Rapid diagnostic test kits
NDA: National drugs Authority
PFP: Private for Profit
PMVs: Patent Medicine Vendors
PPPH: Public Private Partnership for health
RDT: Rapid diagnostic test
SSA: Sub-Saharan Africa
UDHS: Uganda demographic and Health survey
UHC: Universal Health Coverage
UNCST: Uganda National Council for Science and Technology
UNHS: Uganda National Health Survey
UNICEF: United Nationals Children Fund
USD: United States Dollar
WHO: World Health Organization
