# Supplementary material for: Private retail drug shops: what they are, how they operate, and implications for health care delivery in rural Uganda
Source: BMC Health Serv Res. 2018 Jul 9;18:532. doi: 10.1186/s12913-018-3343-z (PMC6038354; doi:10.1186/s12913-018-3343-z)
Supplement: Supplementary file 1 — Drugshop Survey Tool This is the questionnaire that was administered to drug sellers to generate information on the drug shop market. (DOC 216 kb). [file 12913_2018_3343_MOESM1_ESM.doc]

**Drug Shop Client Exit Interview Tool**

**Analysis of private retail drug shops and their potential to implement interventions that improve under-five child health in Uganda**

| *Type* | *NO.* | *Questions and filters* | *Coding categories* | *Code* | *Skip* |
| --- | --- | --- | --- | --- | --- |
| **D** | **0** | **RESPONDENT AGREES TO BE INTERVIEWED** | **Yes**  **No** | **1**  **2** |  |
| **D** | **1** | **RECORD INTERVIEW DATE** | | **Day** | | **Month** | | **Year** | | | --- | --- | --- | --- | --- | --- | |  |  |  |  |  |  | |  |  |

**Interviewer’s Name: Signature:**

**District: Sub-county:**

**Questionnaire Number: Supervisor’s Name:**

|  |  | |  |  |
| --- | --- | --- | --- | --- |
| *Type* | *Questions and filters* | *Coding categories* | *Code* | *Skip* |
| **1** | Did you come to the drug shop today seeking medicines for treatment of a child who is less than five years old? | Yes  No | 1  2 | **If NO**  **XXX** |
| **2** | What symptoms did the child have when you brought them to the drugshop? (check all mentioned) | 1) Fever  2) Cough  3) Rapid/difficult breathing  4) Diarrhea  Other illness (SPECIFY) | **1**  **2**  **3**  **4** |  |
| **3** | How old is the child? | In completed months |  |  |
| **4** | Child’s Gender | Male  Female | 1  2 |  |
| **5** | When did you notice the illness (symptoms) before you decided to come to treatment? | Less than 24 hours  Between 24-48 hours ago  More than 48 hours ago  Do not know | **1**  **2**  **3**  **4** |  |
| **6** | Did you take the sick child anywhere else for treatment before you came to this drug shop? | Yes  No | **1**  **0** |  |
| **7** | If (6 above is) yes, where did you take the child first? | Health center  Hospital Private clinic Community medicine distributor (VHT) Traditional healer  Other drug shop  Others (SPECIFY)………………. | **1**  **2**  **3**  **4**  **5**  **6**  **7** |  |
| **8** | Was the child given any treatment at home before coming to this drug shop? | Yes  No | **1**  **0** |  |
| **9** | If (for 8 above) yes, what did you give? **TICK All FOR EACH SYMPTOM**   | **No** | **Drug** | **Yes** | **No** | | --- | --- | --- | --- | | 1 | Paracetamol |  |  | | 2 | Septrin syrup |  |  | | 3 | Septrin tablet |  |  | | 4 | Amoxycillin syrup |  |  | | 5 | Amoxycillin capsule |  |  | | 6 | Amoxycillin tablet |  |  | | 7 | Antimalarials Fansidar |  |  | | 8 | Antimalarials ACT |  |  | | 9 | Antimalarials Chloroquine |  |  | | 10 | Antimalarials Quinine |  |  | | 11 | Multivitamins |  |  | | 12 | Cough syrup |  |  | | 13 | Oral rehydration therapy |  |  | | 14 | De-worming tablets |  |  | | 15 | Local herbs |  |  | | 16 | Amoxycillin Pink |  |  | | 17 | Amoxycillin Green |  |  | | 18 | ACT yellow |  |  | | 19 | ACT blue |  |  | | 20 | Oral Rehydration Salts + Zinc |  |  | | 21 | Others………………………. |  |  | | | |  |
|  |  |  |  |  |
| **10** | Now, I would like to see the medicines you have purchased **For each medicine seen, fill out details as below.**  **REPEAT for each product bought for FEVER, COUGH/DIFFICULT BREATHING and DIARRHOEA**   | No  . | Drug | Brand name  and manufacturer | dosage | Dosage  formulation (tablets/syrup/ Powder) | Number of  tablets/capsules/  sachets bought | total  amount paid | | --- | --- | --- | --- | --- | --- | --- | | 1 | Paracetamol |  |  |  |  |  | | 2 | Septrin syrup |  |  |  |  |  | | 3 | Septrin tablet |  |  |  |  |  | | 4 | Amoxycillin  syrup |  |  |  |  |  | | 5 | Amoxycillin  capsule |  |  |  |  |  | | 6 | Amoxycillin  Tablet |  |  |  |  |  | | 7 | Antimalarials  Fansidar |  |  |  |  |  | | 8 | Antimalarials  ACT |  |  |  |  |  | | 9 | Antimalarials  Chloroquine |  |  |  |  |  | | 10 | Antimalarials  Quinine |  |  |  |  |  | | 11 | Multivitamins |  |  |  |  |  | | 12 | Cough syrup |  |  |  |  |  | | 13 | Oral rehydration  therapy |  |  |  |  |  | | 14 | De-worming  tablets |  |  |  |  |  | | 15 | Amoxycillin  Pink |  |  |  |  |  | | 16 | Amoxycillin  Green |  |  |  |  |  | | 17 | ACT Yellow |  |  |  |  |  | | 18 | ACT Blue |  |  |  |  |  |  | 19 | Oral  Rehydration Salts + Zinc (ORS/Zinc) |  |  |  |  |  | | --- | --- | --- | --- | --- | --- | --- | | | | |
|  |  |  |  |  |
| **11** | For each of the purchased medicines, ask if the drug seller/pharmacist told the buyer  how to use the medicine including how many times per day; how many tablets each time; total duration of treatment and fill in the table below.   | N  o | Drug | Did the drug  seller give you instructions on how to take this medicine? | How  many times per day? | How  many tablets/s achets each time | How many  tablets/caps ules/  sachets per day | Total  duration of treatmen t  (days) | Appropriateness of  treatment (**to be filled by field supervisor after interview**) | | --- | --- | --- | --- | --- | --- | --- | --- | | 1 | Paracetamol |  |  |  |  |  |  | | 2 | Septrin syrup |  |  |  |  |  |  | | 3 | Septrin tablet |  |  |  |  |  |  | | 4 | Amoxycillin  syrup |  |  |  |  |  |  | | 5 | Amoxycillin  capsule |  |  |  |  |  |  | | 6 | Amoxycillin  tablet |  |  |  |  |  |  | | 7 | Antimalarials  Fansidar |  |  |  |  |  |  | | 8 | Antimalarials  ACT |  |  |  |  |  |  | | 9 | Antimalarials  chloroquine |  |  |  |  |  |  | | 10 | Antimalarials  quinine |  |  |  |  |  |  | | 11 | Multivitamins |  |  |  |  |  |  | | 12 | Cough syrup |  |  |  |  |  |  | | 13 | Oral  rehydration therapy |  |  |  |  |  |  | | 14 | De-worming  tablets |  |  |  |  |  |  | | 15 | Amoxycillin  Pink |  |  |  |  |  |  | | 16 | Amoxycillin  Green |  |  |  |  |  |  | | 17 | ACT Yellow |  |  |  |  |  |  | | 18 | ACT Blue |  |  |  |  |  |  | | 19 | Oral |  |  |  |  |  |  | |  | Rehydration  Salts + Zinc tablets (ORS/Zinc) |  |  |  |  |  |  | | 20 | Zinc tablets  only |  |  |  |  |  |  | | | |  |
|  |  |  |  |  |
| **12** | How were the instructions given? | Orally  Written  Both written and orally | **1**  **2**  **3** |  |
| **13** | Did the drug seller/pharmacist ask you to repeat the instructions? | Yes  No | **1**  **0** |  |
| **14** | Check for mother’s understanding of the instructions (ask care seeker to repeat the instructions written on the medicine envelope | Correctly repeated all instructions  Correctly for some instructions  Incorrectly for most/all | **1**  **2**  **3** |  |
| **15** | What other special instructions were you given? (INTERVIEWER TO WRITE ) |  | | |
| **16** | Did you buy the drugs that had been prescribed for your child? | Yes  No | **1**  **0** |  |
| **17** | If not prescribed, how did you decide what medicine to buy? | Had used it before  Advised by a friend/relative  Advised by the drug seller/pharmacist  I know the drug and asked for it  Other explanation  (Specify)................. | **1**  **2**  **3**  **4** |  |
|  |  |  |  |  |
|  | **Accessibility and Affordability of Drugs** | | |  |
| **18** | After noticing the child was sick, how long did you wait before coming to this shop to buy drugs? | Less than 24 hours  Between 24-48 hours  More than 48 hours  Do not know | **1**  **2**  **3**  **4** |  |
| **19** | What is the reason for choosing this facility?  **(DONOT PROMPT, MULTIPLE RESPONSES ACCEPTED)** | Near distance  Open all the time  Can borrow medicine  Drug seller my Friend  Regular supply of drugs  Good service/customer care  Recommended  Good/trained staff  Other (Specify) | **1**  **2**  **3**  **4**  **5**  **6**  **7**  **8** |  |
| **20** | How long does it take you to walk from your home to this drug shop? | Less than 15 minutes  15-30  30 minutes-1 hour  1-2 hours  more than 2 hours  Do not know | **1**  **2**  **3**  **4**  **5**  **6**  **7** |  |
| **21** | Did you spend any money (on transport) to get here? | Yes  No | **1**  **0** | **Cost if yes** |
| **22** | How long does it take to walk to the nearest health facility? (Clinic, health center, hospital) | Less than 15 mins  15-30mins  30-1 hour  1-2 hours  More than 2 hours  Do not know | **1**  **2**  **3**  **4**  **5**  **6**  **7** |  |
| **23** | Did you buy all the drugs as prescribed/advised? | Yes  No | **1**  **0** |  |
| **24** | If No, Why? | I did not prefer to buy complete dose  I did not have enough money  I have other medicines at home  Other (specify) | **1**  **2**  **3** |  |
| **25** | When buying drugs what determines the amount you buy? | The dosage prescribed  The amount of money I have  The amount advised by friend/relative  The severity of sickness  Other (specify)…………………………….. | **1**  **2**  **3**  **4** |  |
| **26** | How do you rate the prices of drugs in this outlet with respect to your ability to buy them? | They are too expensive  Price are within my reach  I usually find them cheaper  I don’t know | **1**  **2**  **3**  **4** |  |
|  | **Buyer characteristics** | |  |  |
| **27** | How old are you? | In full years |  |  |
| **28** | Gender | Male  Female | **1**  **2** |  |
| **29** | Have you ever attended school? | Yes  No | **1**  **2** |  |
| **30** | What is the highest level of school you attended: primary, secondary or higher? | Primary  O Level  A Level  University  Tertiary | **1**  **2**  **3**  **4**  **5** |  |
| **31** | What is your employment status? | Unemployed  Housewife  Self-employed  Subsistence farmer  Employed by family business/farm  Employed by government/Local authority  Employed in private sector/NGO  Retired  Others  (specify)…………………………………….. | **1**  **2**  **3**  **4**  **5**  **6**  **7**  **8** |  |
| **32** | At this drug shop, what medical investigations have been done on the child? | Rapid Diagnostic Test for malaria  Breathing counted using Respiratory Timer  Temperature taken using a thermometer  No investigations done, just bought drugs | **1**  **2**  **3**  **4** |  |
| **33** | According to you, how severe is the child’s illness? | Very Severe  Moderately Severe  Not severe  Don’t know | **1**  **2**  **3**  **4** |  |
| **34** | Do you have anything else to say that you think may be important for me to know?  ...................................................................................................................................................  ...................................................................................................................................................  ....................................................................................................................................... | | | |
|  |  |  |  |  |
