# Supplementary material for: Private retail drug shops: what they are, how they operate, and implications for health care delivery in rural Uganda
Source: BMC Health Serv Res. 2018 Jul 9;18:532. doi: 10.1186/s12913-018-3343-z (PMC6038354; doi:10.1186/s12913-018-3343-z)
Supplement: Supplementary file 2 — Client Exit Interview Tool This is the questionnaire that was administered to drug shop clients at their exit from the drug shop when they came to purchase medicines or drugs for under-five children, during the study period. (DOC 520 kb). [file 12913_2018_3343_MOESM2_ESM.doc]

**Drug Shop Survey Tool**

**Analysis of private retail drug shops and their potential to implement interventions that improve under-five child health in Uganda**

**Retail Drug shop/ Market survey Tool**

**Drug Shop details**

| Drug shop  Unique ID (B001-B999; M001-M999): |
| --- |
| Sub county: |
| District: Bushenyi (Control)………………………………………………………………..1  Mbarara(Intervention)……………………………………………………………...2 |
| In-charge |
| Contact details  Telephone:  Email: |
| Respondents: |
| Date of interview (dd/mm/yy): |
| Data collector: |
| Study category: Baseline…………………………………………………………………………...1  Evaluation………………………………………………………………………...2 |

| **A** |  |  | | | |
| --- | --- | --- | --- | --- | --- |
|  |  | |  | code |  |
| A1 | Background of owner/manager  *Items listed to the right should not be read out, they are for the data collector to use to code the response; prompts can be made*  Tick one | | Pharmacy technician | 1 |  |
|  |  |  | Clinical officer/Medical assistant | 2 |  |
|  |  |  | Nurse | 3 |  |
|  |  |  | Nursing assistant (trained) | 4 |  |
|  |  |  | Medical doctor | 5 |  |
|  |  |  | Other health profession (specify) | 6 |  |
| A2 | Background of person providing the information (Attendant) | | Pharmacy technician | 1 |  |
|  |  |  | Clinical officer/Medical assistant | 2 |  |
|  |  |  | Nurse | 3 |  |
|  |  |  | Nursing assistant (trained) | 4 |  |
|  |  |  | Medical doctor | 5 |  |
|  |  |  | Other health profession (specify) | 6 |  |
| A3 | How long has the drug shop been operating in complete months? | | |  |  |
| A4 | How many workers including you, work at the drug shop? | | |  |  |
| A5 | Of those working here, how many are: (fill in numbers) | | Pharmacy technician |  |  |
|  |  |  | Nurses |  |  |
|  |  |  | Medical doctor |  |  |
|  |  |  | Midwives |  |  |
|  |  |  | Clinical officers |  |  |
|  |  |  | Nursing officers |  |  |
|  |  |  | Nursing assistants |  |  |
|  |  |  | Others health profession (state) |  |  |
| A6 | How are staff are paid (remunerated) in this drug shop? | | Monthly salary (payment) only | 1 |  |
|  |  |  | Proportion of shop income/month | 2 |  |
|  |  |  | Other incentives (specify) | 3 |  |
| A7 | If monthly payments, on average, how much is a worker paid monthly at this drug shop (In UgShs)? | | 50,000-100,000 | 1 |  |
|  |  |  | 105,000-150,000 | 2 |  |
|  |  |  | 155,000-200,000 | 3 |  |
|  |  |  | 205,000+ | 4 |  |
| A8 | What is the opening and closing hours of this drug shop? (fill in time, 24 hour) | | Opens at |  |  |
|  |  |  | Closes at |  |  |
|  |  |  | Opening hours (enumerator to calculate) |  |  |
| A9 | Does the drug shop open on Sunday to serve patients as well? | | Yes | 1 |  |
|  |  |  | No | 0 |  |
| A10 | Name of the nearest public health facility | |  |  | |
| A11 | Level of care of nearest public health facility | | Regional Referral Hospital | 1 |  |
|  |  |  | District General Hospital | 2 |  |
|  |  |  | HCIV | 3 |  |
|  |  |  | HCIII | 4 |  |
|  |  |  | HCII | 5 |  |
| A12 | How long (in minutes) by walking, does it take (from drug shop) to reach the nearest public health facility? | | |  |  |
| A13 | How long (in minutes) by walking, does it take (from this drug shop) to reach the nearest drug shop? | | |  |  |
| A14 | Does the drug shop keep any records clients served? | | Yes (refer to checklist) | 1 |  |
|  |  |  | No (ask questions below here) | 0 |  |
| A15 | In the last one week, what has been the total number of patients this drug shop has serve | | |  |  |
|  |  |  |  | Don’t know |  |
| A16 | In the last one week, how many of these were children 5 years or below? | | |  |  |
|  |  |  |  | Don’t know |  |
| A17 | In the last one week, how many children were treated for febrile illness? | | |  |  |
|  |  |  |  | Don’t know |  |
| A18 | In the last one week, how many children were treated for coughing or difficulty in breathing? | | |  |  |
|  |  |  |  | Don’t know |  |
| A19 | What items are there for sale in the drug shop?  (INTERVIEWER CAN OBSERVE OR EVEN PROBE IF NECESSARY) | |  | Tick |  |
|  |  |  | Medicines |  |  |
|  |  |  | Food |  |  |
|  |  |  | Soft drinks |  |  |
|  |  |  | Toiletries |  |  |
|  |  |  | Household goods |  |  |
|  |  |  | Alcoholic drinks |  |  |
|  |  |  | Mobile phone airtime |  |  |
|  |  |  | Newspapers |  |  |
|  |  |  | Cosmetics |  |  |
|  |  |  | Cigarettes |  |  |
|  |  |  | Other (specify) |  |  |
| A20 | Do you sell medicines to others to re-sell (other outlets, vendors) | | Yes | 1 |  |
|  |  |  | No | 0 |  |
|  |  |  |  |  |  |
| A23 | How is the decision made on which antimalarial medicines (generally medicines) to stock? | | 1. Medicines recommended by MoH | 1 |  |
|  |  |  | 1. Customer demand | 2 |  |
|  |  |  | 1. Most profitable | 3 |  |
|  |  |  | 1. Recommended by local health professional | 4 |  |
|  |  |  | 1. Special purchase terms offered | 5 |  |
|  |  |  | 1. Lowest priced | 6 |  |
|  |  |  | 1. Drug company/rep influence | 7 |  |
|  |  |  | 1. Influence of the pricing of the same therapeutic group | 8 |  |
|  |  |  | 1. Location of drug shop | 9 |  |
|  |  |  | 1. Country of origin | 10 |  |
|  |  |  | 1. Season disease patterns | 11 |  |
|  |  |  | 1. Clinical conditions of client | 12 |  |
|  |  |  | 1. Other (specify) |  |  |
| A24 | If more than one antimalarial is stocked, how do the staff in the outlet decide which one to sell/dispense | | 1. Medicines recommended by MoH | 1 |  |
|  |  |  | 1. Customer demand | 2 |  |
|  |  |  | 1. Most profitable | 3 |  |
|  |  |  | 1. Recommended by local health professional | 4 |  |
|  |  |  | 1. Special purchase terms offered | 5 |  |
|  |  |  | 1. Lowest priced | 6 |  |
|  |  |  | 1. Personal preference by staff | 7 |  |
|  |  |  | 1. Appearance of patient | 8 |  |
|  |  |  | 1. Fast moving brand | 9 |  |
|  |  |  | 1. Time of the day, week, month | 10 |  |
|  |  |  | 1. Short shelf life | 11 |  |
|  |  |  | 1. Drug company/rep influence | 12 |  |
|  |  |  | 1. Clinical conditions of client | 13 |  |
|  |  |  | Other (state) | | |
| A25 | Do customers ever come with prescriptions to the drug shop? | | Yes | 1 |  |
|  |  |  | No | 2 |  |
| A26 | If yes, where do they often get the prescription from? | | Friends/ Neighbors | 1 |  |
|  |  |  | Nearest Health facilities (staff) | 2 |  |
|  |  |  | Used in last illness episode | 3 |  |
|  |  |  | Others (Specify) |  | |
| A27 | What often brings clients to this shop for? | | To get first aid (basic treatment) | 1 |  |
|  |  |  | Complete treatment(prescriptive) | 2 |  |
| A28 | Approximately how many clients visited your shop last week? | | In total |  |  |
|  |  |  | For fever or malaria treatment |  |  |
|  |  |  | For other drugs |  |  |
| A29 | What type of customers (socioeconomic status) often visit your shop for fever or malaria medicines? | | The poor | 1 |  |
|  |  |  | The well off | 2 |  |
|  |  |  | Both poor and well-off | 3 |  |
|  |  |  | Don’t know | 4 |  |
| A30 | Do you know most of the customers? | | Yes | 1 |  |
|  |  |  | No | 0 |  |
| A31 | Out of patients/customers you served yesterday, how many did not buy a full course of anti-malarial medicine | | |  |  |
|  |  |  |  | Don’t know |  |
| A32 | If the caretaker does not have enough money for the full course of antimalarial treatment, what do you do? | | 1. Sell as many tablets as they have money for | 1 |  |
|  |  |  | 1. Give credit | 2 |  |
|  |  |  | 1. Offer cheaper alternative | 3 |  |
|  |  |  | 1. Refuse to sell | 4 |  |
|  |  |  | 1. Refer elsewhere | 5 |  |
|  |  |  | 1. Other (specify) |  |  |
| A33 | Are all medicines on open display? (vs hidden out of sight) (INTERVIEWER TO PROBE) | | All medicines on display | 1 |  |
|  |  |  | Some are on display | 2 |  |
|  |  |  | None are on display | 3 |  |
| A34 | Have you ever heard of integrated Community Case Management of childhood (iCCM) illnesses? | | Yes | 1 |  |
|  |  |  | No | 0 |  |
|  |  |  | No | 2 |  |
| Reference materials available in the drug shops | | |  |  |  |
| A35 | Uganda Clinical Guidelines | | Yes | 1 |  |
|  |  |  | No | 0 |  |
| A36 | iCCM guidelines | | Yes | 1 |  |
|  |  |  | No | 0 |  |
| A37 | Malaria Treatment charts/posters/algorithms | | Yes | 1 |  |
|  |  |  | No | 0 |  |
| A38 | Pneumonia treatment charts/posters/algorithms | | Yes | 1 |  |
|  |  |  | No | 0 |  |
| A39 | Diarrhoea treatment charts/posters/algorithms | | Yes | 1 |  |
|  |  |  | No | 0 |  |
| A40 | Other charts and guidelines (specify) | | Yes | 1 |  |
|  |  |  | No | 0 |  |
| S | Source of supplies and distribution chain | | | |  |
| S41 | In the last 3 months have you bought any supplies for antimalarials? | | Yes | 1 |  |
|  |  |  | No | 0 |  |
| S42 | Who are the main two suppliers where you get your supplies from? (write names of suppliers for example pharmacies) | |  |  |  |
| S43 | What is the level or type of Suppliers? | | Pharmacy | 1 |  |
|  |  |  | Clinic | 2 |  |
|  |  |  | Drug shop | 3 |  |
|  |  |  | Others (specify) | 4 |  |
|  |  |  |  |  |  |
| S44 | How far are your supplies you’re your drugs shop (KMs) | | Less than 1 km | 1 |  |
|  |  |  | 1. 3km | 2 |  |
|  |  |  | 3+ kms | 3 |  |
|  |  |  | Don’t Know | 9 |  |
| S45 | What are the main reasons why you procure from these suppliers? | | Distance (Convenient) | 1 |  |
|  |  |  | Offer credit facilities | 2 |  |
|  |  |  | Cheap products | 3 |  |
|  |  |  | Trust | 4 |  |
|  |  |  | After sale services (transport) | 5 |  |
| S46 | How do you receive medicines from your supplier? | | Supplier delivers them | 1 |  |
|  |  |  | I Pick them from supplier stores | 2 |  |
|  |  |  | Others (Specify) | 3 |  |
|  |  | |  |  |  |
| S47 | If picked by yourself or delivered, what is the mode of delivery? | | By car  By bicycle  By motorcycle  Others (specify) | 1  2  3  4……… |  |
| S48 | Do you receive any medicines from your supplier on credit? | | Yes  No  Don’t Know | 1  0  9 |  |
|  |  | |  |  |  |
|  | Regulation | | |  |  |
| R49 | Is this shop/business registered and licensed? | | Yes | 1 |  |
|  |  |  | No | 0 |  |
| R50 | If yes, how long did it take to be registered, when the process is started? | | One week | 1 |  |
|  |  |  | Less than one month | 2 |  |
|  |  |  | More than one month | 3 |  |
| R51 | How much did you pay for the license (Ugshs) | | (write amount in box) |  |  |
|  |  |  | Don’t know | 9 |  |
| R52 | Has any health inspector visited you in the last six months? | | Yes | 1 |  |
|  |  |  | No | 0 |  |
| R53 | When was the most recent inspector visit (month)? | |  |  | |
| R54 | What was the most recent supervision focused on? (WRITE FEW ISSUES) | |  | | |
|  |  | | | |  |
| M | Marketing and sales | | | |  |
| M55 | Do you market (advertise) any of your products or services? | | Yes  No | 1  0 |  |
| M56 | If yes, which of the following methods do you use? | | Posters  Special displays  Discounts  Credit  Radio spots  Others  (Specify)……………………….. | 1  2  3  4  5  6 |  |
| M57 | What drugs do most patients want for treatment of malaria? | | (MENTION) |  |  |
| M58 | Of the antimalarial products you have in stock, which product have you sold the most dozes in the last one month? | | (MENTION) | | |
| M59 | In your opinion, what is the most effective antimalarial product of those available on the market? | | (MENTION) |  |  |
| M60 | Do you always sell clients the product/ drugs they want? | | Yes  No | 1  0 |  |
| M61 | Let’s focus on specific drugs (ACTs), how many doses do you sell in a typical month? (select number of drugs/products) | | Drug A: (name) |  |  |
|  |  |  | Drug B (Name) |  |  |
|  |  |  | Drug C (name) |  |  |
|  |  |  | Drug D (name) |  |  |
|  |  |  | Drug E (Name) |  |  |
|  |  | |  | Purchase price per doze | Selling price per doze |
| M61 | Let’s focus on specific drugs, how much of mark-up do you receive on each doze (ACTs)? | | Drug A: (name) |  |  |
|  |  |  | Drug B (Name) |  |  |
|  |  |  | Drug C (name) |  |  |
|  |  |  | Drug D (name) |  |  |
|  |  |  | Drug E (Name) |  |  |
|  | Competition | | |  |  |
| C61 | How many drug shops are within this location/ local Town? | | (Write Number) |  |  |
| C62  C63 | Which places do you believe are your main competitors for customers? | | Other drug shops | 1 |  |
|  |  |  | Health centers/clinics | 2 |  |
|  |  |  | Traditional Healers | 3 |  |
|  |  |  | Others (specify) |  |  |
| C64  C64 | Why do you consider them to be your competitors? | | Their prices are low | 1 |  |
|  |  |  | Offer other additional services | 2 |  |
|  |  |  | Have qualified staff | 3 |  |
|  |  |  | Good customer care | 4 |  |
|  |  |  | Offer credit | 5 |  |
|  |  |  | Free services | 6 |  |
|  |  |  | Have been in business for long | 7 |  |
|  |  |  | Close proximity | 8 |  |
|  |  |  | Others (Specify) |  |  |

**Management/treatment and dispensing practices**

**Observe 5 patients (records if they exist) received at the drug shop**

| **No** | **Enter yes (Y), no (N) or a number, regarding the contents of the patient’s prescription** | Child | | | | |
| --- | --- | --- | --- | --- | --- | --- |
|  |  | 1 | 2 | 3 | 4 | 5 |
| B1 | Patient’s/child’s age determined |  |  |  |  |  |
|  | B1i Age of patient/child in years |  |  |  |  |  |
| B2 | Patient’s/child’s weight determined |  |  |  |  |  |
|  | Weight of patient in kilograms |  |  |  |  |  |
| B3 | Asked mother/caretaker about child’s problem |  |  |  |  |  |
| B4 | Checked/assessed child for general danger signs |  |  |  |  |  |
| B5 | Asks the mother/caretaker about four main symptoms |  |  |  |  |  |
|  | 1. Fever |  |  |  |  |  |
|  | 1. Coughing or difficulty in breathing |  |  |  |  |  |
|  | 1. Diarrhea |  |  |  |  |  |
|  | 1. Ear problem |  |  |  |  |  |
| B6 | Following diagnostics used to assess patient |  |  |  |  |  |
|  | 1. Thermometer |  |  |  |  |  |
|  | 1. Malaria Rapid diagnostics |  |  |  |  |  |
|  | 1. Respiratory rate meter |  |  |  |  |  |
|  | 1. Other (specify) |  |  |  |  |  |
|  | **Medicines given** |  |  |  |  |  |
| B7 | Antimalarial medicine (s) prescribed (code) |  |  |  |  |  |
| B8 | Antibiotic medicine (s) prescribed |  |  |  |  |  |
| B9 | ORS (s) prescribed |  |  |  |  |  |
| B10 | Number of ORS sachets given |  |  |  |  |  |
| B11 | Counsel on how to reconstitute and administer |  |  |  |  |  |
| B12 | Total number of medicines given to patient |  |  |  |  |  |
|  | **Dispensing** |  |  |  |  |  |
| B13 | Used appropriate packaging[[1]](#footnote-2) |  |  |  |  |  |
| B14 | If Yes (Y) indicate whether prepackaged |  |  |  |  |  |
| B15 | Used appropriate labeling[[2]](#footnote-3) |  |  |  |  |  |
| B16 | Identified the medication for the patient |  |  |  |  |  |
| B17 | Explains the method of administration |  |  |  |  |  |
| B18 | Explained how often to take the medicines |  |  |  |  |  |
| B19 | Explained the duration of therapy |  |  |  |  |  |
| B20 | Assessed patient’s understanding of instructions |  |  |  |  |  |
| B21 | Was complete dosage of antimalarial dispensed? |  |  |  |  |  |

***Codes for antimalarial medicine:*** *1 = Artemether/Lumefantrine, 2 = Artesunate + Amodiaquine, 3 = Quinine, 4 = Chloroquine, 5 = Sulfadoxine/Pyrimethamine, 6 = Other ACT, 7 = Other non-ACT*

| **px** | **Costs/Pricing** | | | |
| --- | --- | --- | --- | --- |
| P1 | How do you determine the prices on which to sell the drugs or offer services? | **(You can write a number of reasons)** | | |
| P2 | What determines how much to dispense for the patient at your drugs shop? | Patient’s finances |  |  |
|  |  | patient’s preference |  |  |
|  |  | prescribed dosage |  |  |
|  |  | others |  |  |
| P3 | Do the patients pay for diagnostic / testing services at your drugs shop? | Yes |  |  |
|  |  | No |  |  |
| P4 | If yes, how much are they required to pay? |  |  |  |
| P5 | Do you often charge them all that is required to be paid? | Yes |  |  |
|  |  | No |  |  |
| P6 | If no, what determines what amounts you charge them? | **(You can write possible factors)** | | |

**Health information** management systems

| E1 | How do you get information about drugs in terms of new drugs, dosages, treatment guidelines, side-effects? | On Radio | 1 |  |
| --- | --- | --- | --- | --- |
|  |  | During Training | 2 |  |
|  |  | Drug companies come over | 3 |  |
|  |  | District Health office | 4 |  |
|  |  | Fellow health workers | 5 |  |
|  |  | Others (specify) |  |  |
| E2 | Have you had any training in the last six months about dispensing drugs/medicines? | Yes | 1 |  |
|  |  | No | 0 |  |
| E3 | Do you have any system for collecting and managing patient records? | Yes | 1 |  |
|  |  | No | 0 |  |
| E4 | If yes, is it | Paper-based | 1 |  |
|  |  | Computer-based | 2 |  |
|  |  | Both | 3 |  |
| E5 | Does it record the following? |  |  |  |
|  | 1. Patient identity | Yes | 1 |  |
|  |  | No | 0 |  |
|  | 1. Diagnosis | Yes | 1 |  |
|  |  | No | 0 |  |
|  | 1. Medicine given | Yes | 1 |  |
|  |  | No | 0 |  |
|  | 1. Patients referred | Yes | 1 |  |
|  |  | No | 0 |  |
| E6 | Do you have any system for monitoring drug stock level? | Yes | 1 |  |
|  |  | No | 0 |  |
| E7 | If yes, is it | Paper-based | 1 |  |
|  |  | Computer-based | 2 |  |
|  |  | Both | 3 |  |
| E8 | Does it capture the following information? |  |  |  |
|  | 1. Physical stock count | Yes | 1 |  |
|  |  | No | 0 |  |
|  | 1. Quantities received | Yes | 1 |  |
|  |  | No | 0 |  |
|  | 1. Quantities consumed per month | Yes | 1 |  |
|  |  | No | 0 |  |
|  | 1. Stock outs | Yes | 1 |  |
|  |  | No | 0 |  |
|  | 1. Expired stock | Yes | 1 |  |
|  |  | No | 0 |  |
| E9 | Do you have any system for monitoring and reporting your patients who do not respond to treatment given? | Yes | 1 |  |
|  |  | No | 0 |  |
| E10 | If yes, how do you use the information? |  |  |  |

**Access to quality** medicines

| True=1, False = 0 | Adequate storage conditions and handling of medicines – checklist for the outlet and the area where the medicines are stored | Sales/dispensing and storage area [A] |
| --- | --- | --- |
|  | 1. There is a method in place to control temperature e.g. roof and ceiling with space between them in hot climates   (1 = there is a method) |  |
|  | 1. There are windows that can be opened or there are air vents   (1 = there are windows) |  |
|  | 1. No direct sunlight can enter the area where the medicines are stored (1=no sunlight) |  |
|  | 1. Area free from moisture e.g. leaking ceiling, roof, drains, taps   (1= no moisture) |  |
|  | 1. Medicines are not stored directly on the floor   1=not on floor |  |
|  | 1. There is evidence of pests in the area   (1=no pests) |  |
|  | 1. Loose tablets/capsules are not manipulated by naked hand   (1=not handled) |  |
|  | 1. Premises can be secured to prevent theft   (1= secure) |  |
|  | Sum of A |  |
|  | % adequate storage conditions = [A]/8*100 |  |

***Drug shop exit Tool for care-takers***

**Study Title:** Analysis of private retail drug shops and their potential to implement interventions that improve under-five child health in Uganda

| *Type* | *NO.* | *Questions and filters* | *Coding categories* | *Code* | *Skip* |
| --- | --- | --- | --- | --- | --- |
| **D** | **0** | **RESPONDENT AGREES TO BE INTERVIEWED** | **Yes**  **No** | **1**  **2** |  |
| **D** | **1** | **RECORD INTERVIEW DATE** | | **Day** | | **Month** | | **Year** | | | --- | --- | --- | --- | --- | --- | |  |  |  |  |  |  | |  |  |

**Interviewer’s Name: Signature:**

**District: Sub-county:**

**Questionnaire Number: Supervisor’s Name:**

|  |  | |  |  |
| --- | --- | --- | --- | --- |
| *Type* | *Questions and filters* | *Coding categories* | *Code* | *Skip* |
| **1** | Did you come to the drug shop today seeking medicines for treatment of a child who is less than five years old? | Yes  No | 1  2 | **If NO**  **XXX** |
| **2** | What symptoms did the child have when you brought them to the drugshop? (check all mentioned) | 1) Fever  2) Cough  3) Rapid/difficult breathing  4) Diarrhea  Other illness (SPECIFY) | **1**  **2**  **3**  **4** |  |
| **3** | How old is the child? | In completed months |  |  |
| **4** | Child’s Gender | Male  Female | 1  2 |  |
| **5** | When did you notice the illness (symptoms) before you decided to come to treatment? | Less than 24 hours  Between 24-48 hours ago  More than 48 hours ago  Do not know | **1**  **2**  **3**  **4** |  |
| **6** | Did you take the sick child anywhere else for treatment before you came to this drug shop? | Yes  No | **1**  **0** |  |
| **7** | If (6 above is) yes, where did you take the child first? | Health center  Hospital Private clinic Community medicine distributor (VHT) Traditional healer  Other drug shop  Others (SPECIFY)………………. | **1**  **2**  **3**  **4**  **5**  **6**  **7** |  |
| **8** | Was the child given any treatment at home before coming to this drug shop? | Yes  No | **1**  **0** |  |
| **9** | If (for 8 above) yes, what did you give? **TICK All FOR EACH SYMPTOM**   | **No** | **Drug** | **Yes** | **No** | | --- | --- | --- | --- | | 1 | Paracetamol |  |  | | 2 | Septrin syrup |  |  | | 3 | Septrin tablet |  |  | | 4 | Amoxycillin syrup |  |  | | 5 | Amoxycillin capsule |  |  | | 6 | Amoxycillin tablet |  |  | | 7 | Antimalarials Fansidar |  |  | | 8 | Antimalarials ACT |  |  | | 9 | Antimalarials Chloroquine |  |  | | 10 | Antimalarials Quinine |  |  | | 11 | Multivitamins |  |  | | 12 | Cough syrup |  |  | | 13 | Oral rehydration therapy |  |  | | 14 | De-worming tablets |  |  | | 15 | Local herbs |  |  | | 16 | Amoxycillin Pink |  |  | | 17 | Amoxycillin Green |  |  | | 18 | ACT yellow |  |  | | 19 | ACT blue |  |  | | 20 | Oral Rehydration Salts + Zinc |  |  | | 21 | Others………………………. |  |  | | | |  |
|  |  |  |  |  |
| **10** | Now, I would like to see the medicines you have purchased **For each medicine seen, fill out details as below.**  **REPEAT for each product bought for FEVER, COUGH/DIFFICULT BREATHING and DIARRHOEA**   | No  . | Drug | Brand name  and manufacturer | dosage | Dosage  formulation (tablets/syrup/ Powder) | Number of  tablets/capsules/  sachets bought | total  amount paid | | --- | --- | --- | --- | --- | --- | --- | | 1 | Paracetamol |  |  |  |  |  | | 2 | Septrin syrup |  |  |  |  |  | | 3 | Septrin tablet |  |  |  |  |  | | 4 | Amoxycillin  syrup |  |  |  |  |  | | 5 | Amoxycillin  capsule |  |  |  |  |  | | 6 | Amoxycillin  Tablet |  |  |  |  |  | | 7 | Antimalarials  Fansidar |  |  |  |  |  | | 8 | Antimalarials  ACT |  |  |  |  |  | | 9 | Antimalarials  Chloroquine |  |  |  |  |  | | 10 | Antimalarials  Quinine |  |  |  |  |  | | 11 | Multivitamins |  |  |  |  |  | | 12 | Cough syrup |  |  |  |  |  | | 13 | Oral rehydration  therapy |  |  |  |  |  | | 14 | De-worming  tablets |  |  |  |  |  | | 15 | Amoxycillin  Pink |  |  |  |  |  | | 16 | Amoxycillin  Green |  |  |  |  |  | | 17 | ACT Yellow |  |  |  |  |  | | 18 | ACT Blue |  |  |  |  |  |  | 19 | Oral  Rehydration Salts + Zinc (ORS/Zinc) |  |  |  |  |  | | --- | --- | --- | --- | --- | --- | --- | | | | |
|  |  |  |  |  |
| **11** | For each of the purchased medicines, ask if the drug seller/pharmacist told the buyer  how to use the medicine including how many times per day; how many tablets each time; total duration of treatment and fill in the table below.   | N  o | Drug | Did the drug  seller give you instructions on how to take this medicine? | How  many times per day? | How  many tablets/s achets each time | How many  tablets/caps ules/  sachets per day | Total  duration of treatmen t  (days) | Appropriateness of  treatment (**to be filled by field supervisor after interview**) | | --- | --- | --- | --- | --- | --- | --- | --- | | 1 | Paracetamol |  |  |  |  |  |  | | 2 | Septrin syrup |  |  |  |  |  |  | | 3 | Septrin tablet |  |  |  |  |  |  | | 4 | Amoxycillin  syrup |  |  |  |  |  |  | | 5 | Amoxycillin  capsule |  |  |  |  |  |  | | 6 | Amoxycillin  tablet |  |  |  |  |  |  | | 7 | Antimalarials  Fansidar |  |  |  |  |  |  | | 8 | Antimalarials  ACT |  |  |  |  |  |  | | 9 | Antimalarials  chloroquine |  |  |  |  |  |  | | 10 | Antimalarials  quinine |  |  |  |  |  |  | | 11 | Multivitamins |  |  |  |  |  |  | | 12 | Cough syrup |  |  |  |  |  |  | | 13 | Oral  rehydration therapy |  |  |  |  |  |  | | 14 | De-worming  tablets |  |  |  |  |  |  | | 15 | Amoxycillin  Pink |  |  |  |  |  |  | | 16 | Amoxycillin  Green |  |  |  |  |  |  | | 17 | ACT Yellow |  |  |  |  |  |  | | 18 | ACT Blue |  |  |  |  |  |  | | 19 | Oral |  |  |  |  |  |  | |  | Rehydration  Salts + Zinc tablets (ORS/Zinc) |  |  |  |  |  |  | | 20 | Zinc tablets  only |  |  |  |  |  |  | | | |  |
|  |  |  |  |  |
| **12** | How were the instructions given? | Orally  Written  Both written and orally | **1**  **2**  **3** |  |
| **13** | Did the drug seller/pharmacist ask you to repeat the instructions? | Yes  No | **1**  **0** |  |
| **14** | Check for mother’s understanding of the instructions (ask care seeker to repeat the instructions written on the medicine envelope | Correctly repeated all instructions  Correctly for some instructions  Incorrectly for most/all | **1**  **2**  **3** |  |
| **15** | What other special instructions were you given? (INTERVIEWER TO WRITE ) |  | | |
| **16** | Did you buy the drugs that had been prescribed for your child? | Yes  No | **1**  **0** |  |
| **17** | If not prescribed, how did you decide what medicine to buy? | Had used it before  Advised by a friend/relative  Advised by the drug seller/pharmacist  I know the drug and asked for it  Other explanation  (Specify)................. | **1**  **2**  **3**  **4** |  |
|  |  |  |  |  |
|  | **Accessibility and Affordability of Drugs** | | |  |
| **18** | After noticing the child was sick, how long did you wait before coming to this shop to buy drugs? | Less than 24 hours  Between 24-48 hours  More than 48 hours  Do not know | **1**  **2**  **3**  **4** |  |
| **19** | What is the reason for choosing this facility?  **(DONOT PROMPT, MULTIPLE RESPONSES ACCEPTED)** | Near distance  Open all the time  Can borrow medicine  Drug seller my Friend  Regular supply of drugs  Good service/customer care  Recommended  Good/trained staff  Other (Specify) | **1**  **2**  **3**  **4**  **5**  **6**  **7**  **8** |  |
| **20** | How long does it take you to walk from your home to this drug shop? | Less than 15 minutes  15-30  30 minutes-1 hour  1-2 hours  more than 2 hours  Do not know | **1**  **2**  **3**  **4**  **5**  **6**  **7** |  |
| **21** | Did you spend any money (on transport) to get here? | Yes  No | **1**  **0** | **Cost if yes** |
| **22** | How long does it take to walk to the nearest health facility? (Clinic, health center, hospital) | Less than 15 mins  15-30mins  30-1 hour  1-2 hours  More than 2 hours  Do not know | **1**  **2**  **3**  **4**  **5**  **6**  **7** |  |
| **23** | Did you buy all the drugs as prescribed/advised? | Yes  No | **1**  **0** |  |
| **24** | If No, Why? | I did not prefer to buy complete dose  I did not have enough money  I have other medicines at home  Other (specify) | **1**  **2**  **3** |  |
| **25** | When buying drugs what determines the amount you buy? | The dosage prescribed  The amount of money I have  The amount advised by friend/relative  The severity of sickness  Other (specify)…………………………….. | **1**  **2**  **3**  **4** |  |
| **26** | How do you rate the prices of drugs in this outlet with respect to your ability to buy them? | They are too expensive  Price are within my reach  I usually find them cheaper  I don’t know | **1**  **2**  **3**  **4** |  |
|  | **Buyer characteristics** | |  |  |
| **27** | How old are you? | In full years |  |  |
| **28** | Gender | Male  Female | **1**  **2** |  |
| **29** | Have you ever attended school? | Yes  No | **1**  **2** |  |
| **30** | What is the highest level of school you attended: primary, secondary or higher? | Primary  O Level  A Level  University  Tertiary | **1**  **2**  **3**  **4**  **5** |  |
| **31** | What is your employment status? | Unemployed  Housewife  Self-employed  Subsistence farmer  Employed by family business/farm  Employed by government/Local authority  Employed in private sector/NGO  Retired  Others  (specify)…………………………………….. | **1**  **2**  **3**  **4**  **5**  **6**  **7**  **8** |  |
| **32** | At this drug shop, what medical investigations have been done on the child? | Rapid Diagnostic Test for malaria  Breathing counted using Respiratory Timer  Temperature taken using a thermometer  No investigations done, just bought drugs | **1**  **2**  **3**  **4** |  |
| **33** | According to you, how severe is the child’s illness? | Very Severe  Moderately Severe  Not severe  Don’t know | **1**  **2**  **3**  **4** |  |
| **34** | Do you have anything else to say that you think may be important for me to know?  ...................................................................................................................................................  ...................................................................................................................................................  ....................................................................................................................................... | | | |
|  |  |  |  |  |

1. *Appropriate packaging: drugs are packaged in clean dry sealable envelopes or containers.* [↑](#footnote-ref-2)
2. *Appropriate labeling: drugs are clearly labeled with correct drug name, dose, duration of therapy and number of dosing units dispensed.* [↑](#footnote-ref-3)
